# Competitive Endogenous RNA Landscape in Epstein-Barr Virus Associated Nasopharyngeal Carcinoma

**DOI:** 10.3389/fcell.2021.782473

**Published:** 2021-11-04

**Authors:** Xiandong Lin, Steven Wang, Keyu Lin, Jingfeng Zong, Qianlan Zheng, Ying Su, Tao Huang

**Affiliations:** ^1^ Laboratory of Radiation Oncology and Radiobiology, Fujian Medical University Cancer Hospital and Fujian Cancer Hospital, Fuzhou, China; ^2^ Fujian Provincial Key Laboratory of Translational Cancer Medicine, Fuzhou, China; ^3^ Department of Biological Sciences, Columbia University, New York, NY, United States; ^4^ Department of Radiotherapy, Fujian Medical University Cancer Hospital and Fujian Cancer Hospital, Fuzhou, China; ^5^ Bio-Med Big Data Center, Shanghai Institute of Nutrition and Health, University of Chinese Academy of Sciences, Chinese Academy of Sciences, Shanghai, China

**Keywords:** nasopharyngeal carcinoma (NCP), epstein-barr virus (EBV), circRNA, miRNA, network

## Abstract

Non-coding RNAs have been shown to play important regulatory roles, notably in cancer development. In this study, we investigated the role of microRNAs and circular RNAs in Nasopharyngeal Carcinoma (NPC) by constructing a circRNA-miRNA-mRNA co-expression network and performing differential expression analysis on mRNAs, miRNAs, and circRNAs. Specifically, the Epstein-Barr virus (EBV) infection has been found to be an important risk factor for NPC, and potential pathological differences may exist for EBV+ and EBV- subtypes of NPC. By comparing the expression profile of non-cancerous immortalized nasopharyngeal epithelial cell line and NPC cell lines, we identified differentially expressed coding and non-coding RNAs across three groups of comparison: cancer vs. non-cancer, EBV+ vs. EBV- NPC, and metastatic vs. non-metastatic NPC. We constructed a ceRNA network composed of mRNAs, miRNAs, and circRNAs, leveraging co-expression and miRNA target prediction tools. Within the network, we identified the regulatory ceRNAs of *CDKN1B, ZNF302, ZNF268,* and *RPGR*. These differentially expressed axis, along with other miRNA-circRNA pairs we identified through our analysis, helps elucidate the genetic and epigenetic changes central to NPC progression, and the differences between EBV+ and EBV- NPC.

## Introduction

Nasopharyngeal carcinoma (NPC) is an Epstein-Barr virus (EBV) associated malignancy with a characteristic geographical distribution. Globally, NPC is a rare condition, with less than one case per 100,000 people per year. However, the occurrence of NPC is much more common among the populations in Southern China and Southeast Asia, with up to 25–50 cases per 100,000 people per year (Jain et al., 2016). Ethnic Chinese born in North America develop NPC less frequently compared to those in Southern China, implying that both genetic susceptibility and environmental factors contribute to the development of NPC (Buell, 1974). Ample evidence shown that infection of EBV is a risk factor for NPC (Tsao et al., 2017; Chan et al., 2018). For instance, EBV genome and gene products are detected in virtually all tumors in NPC-endemic areas. Increased levels of IgA antibodies against EBV antigens, among other EBV-related biomarkers, have been used for early detection and screening for NPC in a few high-incidence areas (Shotelersuk et al., 2000; Li et al., 2018; Wu et al., 2018). Nevertheless, while many risk factors have been established, our understanding of the molecular regulatory mechanisms that lead to the development of NPC is limited.

The role of various non-coding RNAs in the regulation of many biological pathways and functions have been widely explored and verified in recent years. MicroRNAs (miRNAs) are non-coding RNAs around 22 nucleotides in length that play important regulatory roles, specifically by inhibiting gene expression through cleavage of mRNA or translational repression (Bartel, 2004). In addition to mRNAs, miRNAs interact with any target RNAs that contain complementary sites known as miRNA response elements (MREs). Since miRNAs can bind to multiple targets, the ceRNA hypothesis was proposed, stating that these target RNAs compete for a limited amount of miRNA (Salmena et al., 2011). In other words, the amount of ceRNAs can collectively impact the degree to which miRNAs regulate gene expression.

Circular RNAs (circRNAs) is a type of non-coding RNA structured as a covalently-bonded closed continuous loop, where the 5’-cap and 3’-poly-A tail are joined together. Studies have found circRNAs to play important regulatory roles in NPC growth and metastasis (Chen et al., 2019; Guo et al., 2019; Zhu et al., 2019; Yang et al., 2020). It is widely accepted that circRNAs inhibit target miRNA activity through a miRNA sponge mechanism, which in turn results in an upregulation of target gene expression (Panda, 2018; Zu et al., 2020). A study by Zhu et al. showed that highly expressed circ-ZNF609 absorbs microRNA-150-5p to upregulate Sp1 expression, which in turn promotes the proliferation and metastatic ability of NPC (Zhu et al., 2019). Another study by Yang et al. utilized a circRNA-miRNA-target gene network to reveal potential mechanism between circKITLG and miR-3198 (Yang et al., 2020). Furthermore, studies have shown the circRNA expression is tissue specific (Memczak et al., 2013; You et al., 2015). In short, the interactions between circRNAs and miRNAs have significant influence on key genes, and as a consequence affect the development and progression of cancer (Peng and Croce, 2016; Hirono et al., 2019; Hoey et al., 2019; Hong et al., 2019; Liu et al., 2020).

To analyze and illustrate this complicated collection of interactions, we constructed a circRNA-miRNA-mRNA network. We also performed differential expression (DE) analysis on three groups of comparison: cancerous vs. non-cancerous, EBV+ vs. EBV- cell lines, and metastatic vs. non-metastatic samples. Using these results, we identified axes of circRNA-miRNA-mRNA that are differentially expressed in each group of comparison. We identified 6 differentially expressed circRNA-miRNA-mRNA axes between EBV+ and EBV- cell lines, out of which we highlighted the *hsa_circ_0008129/miR-221-3p/CDKN1B* axis. Through this research, we identified several potential ceRNA axes that regulate NPC progression or EBV associated traits in NPC. These ceRNA pathways can help better understand the molecular landscape of NPC, and help guide therapeutic efforts.

## Results

### Overview of Computational Approach

**FIGURE 1:**
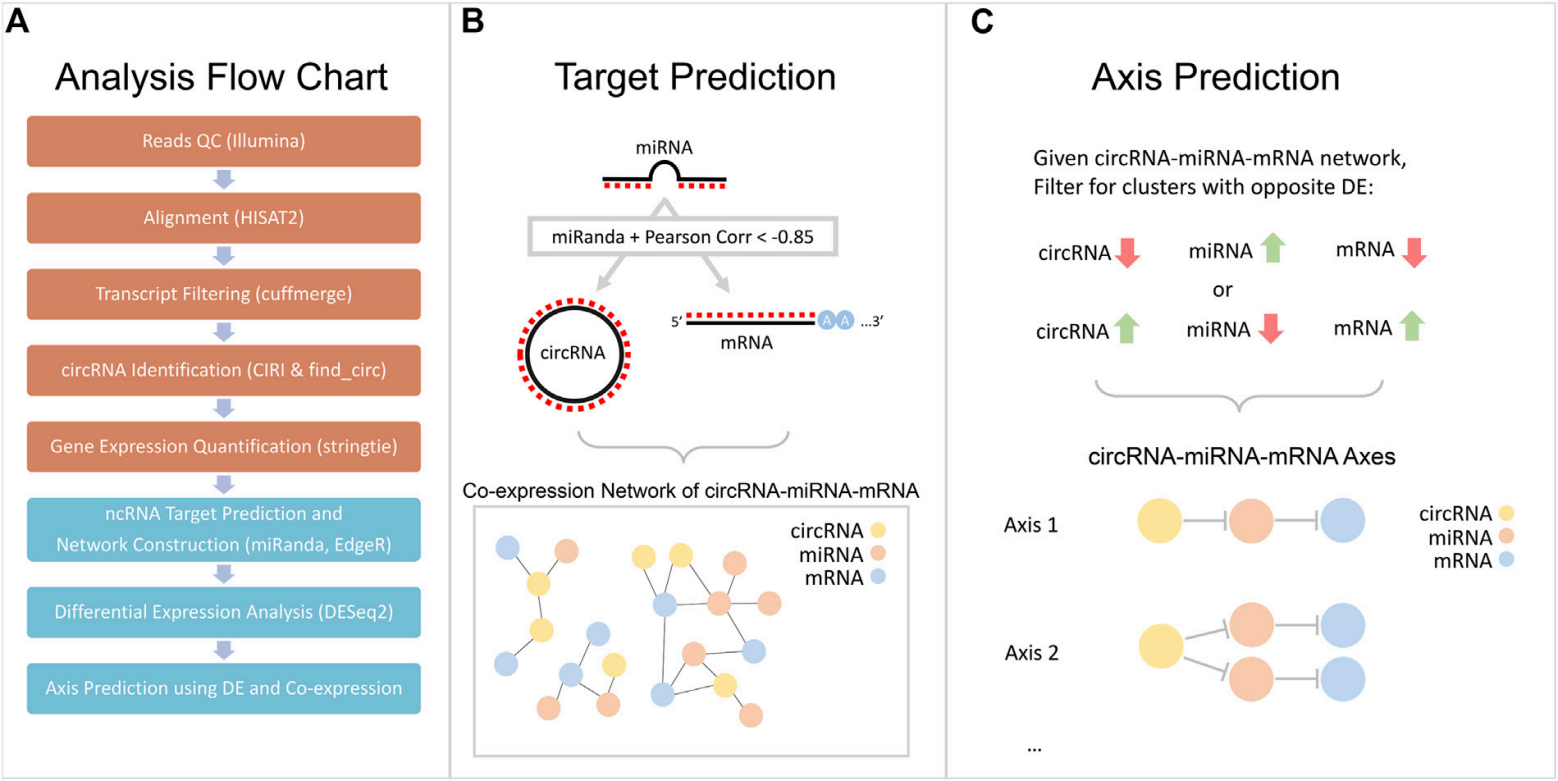
Flowchart of computational workflow. **(A)** Four cell lines and two patient samples were sequenced and processed to obtain an expression quantification for all three types of RNAs: circRNA, miRNA, and mRNA. **(B)** A graphical illustration of the construction of a co-expression network using Pearson correlation and miRanda target prediction. **(C)** A graphical illustration of axis prediction method, utilizing DE results and co-expression network.

We obtained the mRNA, miRNA, and circRNA expression profile for four cancer cell lines and four patient samples. We then performed differential expression (DE) analysis and constructed RNA interaction networks to isolate circRNA-miRNA-mRNA axes of interest. Simultaneously, we performed functional enrichment of RNAs of interest to reveal relevant pathways in the pathology of NPC. We provided a graphical outline of the computational workflow in Figure 1. Lastly, we cross-referenced our analysis results with two publicly available NPC RNA-seq datasets, GSE143797 (Yang et al., 2020) and GSE118721 (Lin et al., 2018), to highlight intersecting DE-RNAs.

### Differential Expression Analysis and Functional Enrichment Analysis

**FIGURE 2:**
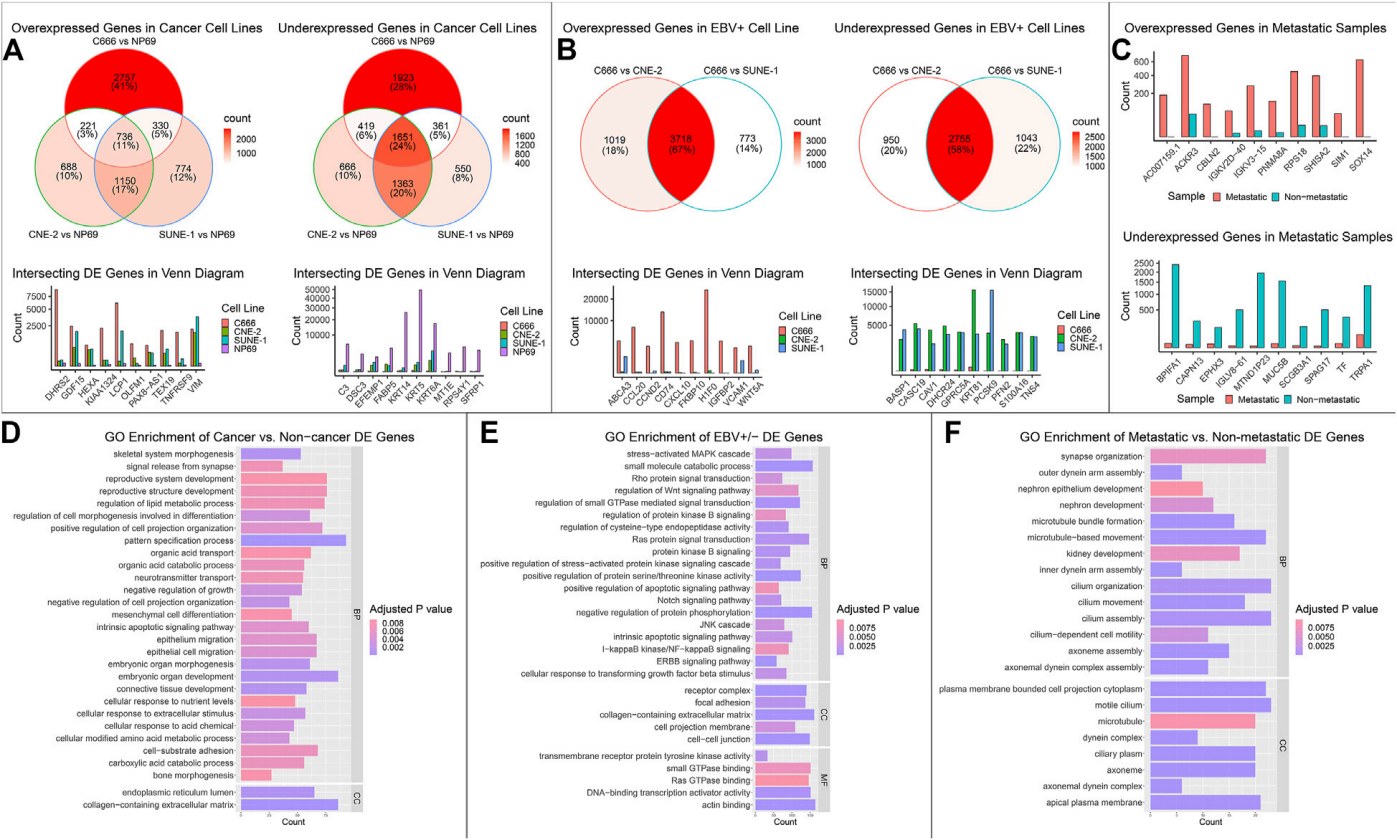
Overview of mRNA landscape across comparisons. The differential expression (DE) results for mRNAs across the three groups of comparison: cancer vs. non-cancer, EBV + vs. EBV-, and metastatic vs non-metastatic, were integrated into one figure. **(A)** The cancer vs. non-cancer group consisted of three sets of DE analysis: NP69 (non-cancer) vs. C666, CNE-2, and SUNE-1 (cancer). The expression of top DE genes that intersected all three sets of DE analysis was plotted. **(B)** The EBV+ vs. EBV- group consisted of two sets of DE analysis: C666 (EBV+) vs. CNE-2, SUNE-1 (EBV-). The expression of intersecting DE genes was plotted. **(C)** Since there was only one set of DE analysis, the expression of top DE genes was plotted. **(D–F)** The intersecting DE genes in each comparison were functionally enriched using the GO database, and top enriched pathways were plotted. The x-axis value represented the gene count in the corresponding pathway, and the color of the bar represented the adjusted *p* value of the over representation test.

**FIGURE 3:**
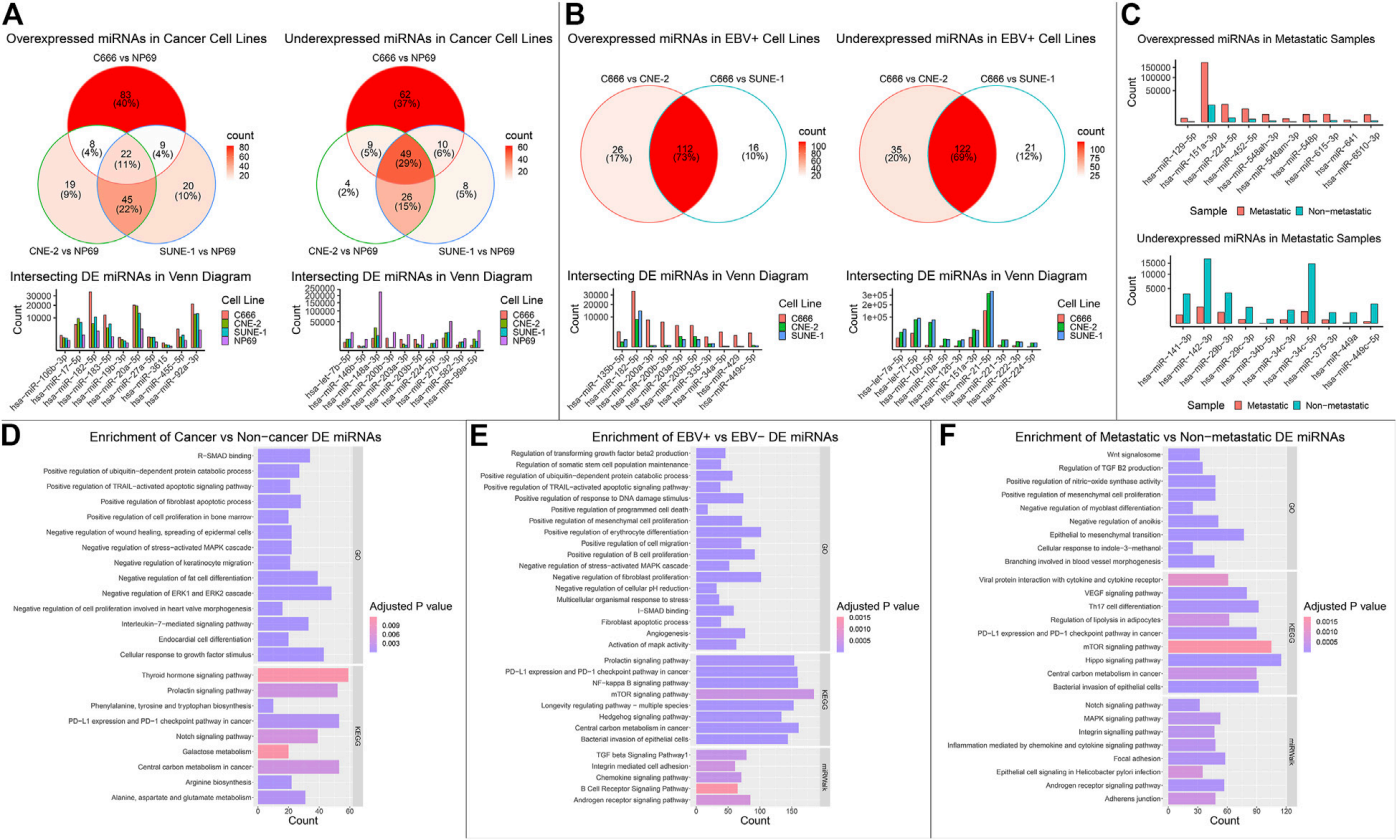
Overview of miRNA landscape across comparisons. The differential expression (DE) results for miRNAs across the three groups of comparison were integrated into one figure. **(A–C)** The expression of top intersecting DE-miRNA in each comparison was plotted. **(D–F)** The intersecting DE-miRNAs in each comparison were functionally enriched using the miEAA website, surveying across GO, KEGG, and miRWalk databases. The top enriched pathways were plotted. The x-axis value represented the gene count in the corresponding pathway, and the color of the bar represented the adjusted *p* value of the over representation test.

**FIGURE 4:**
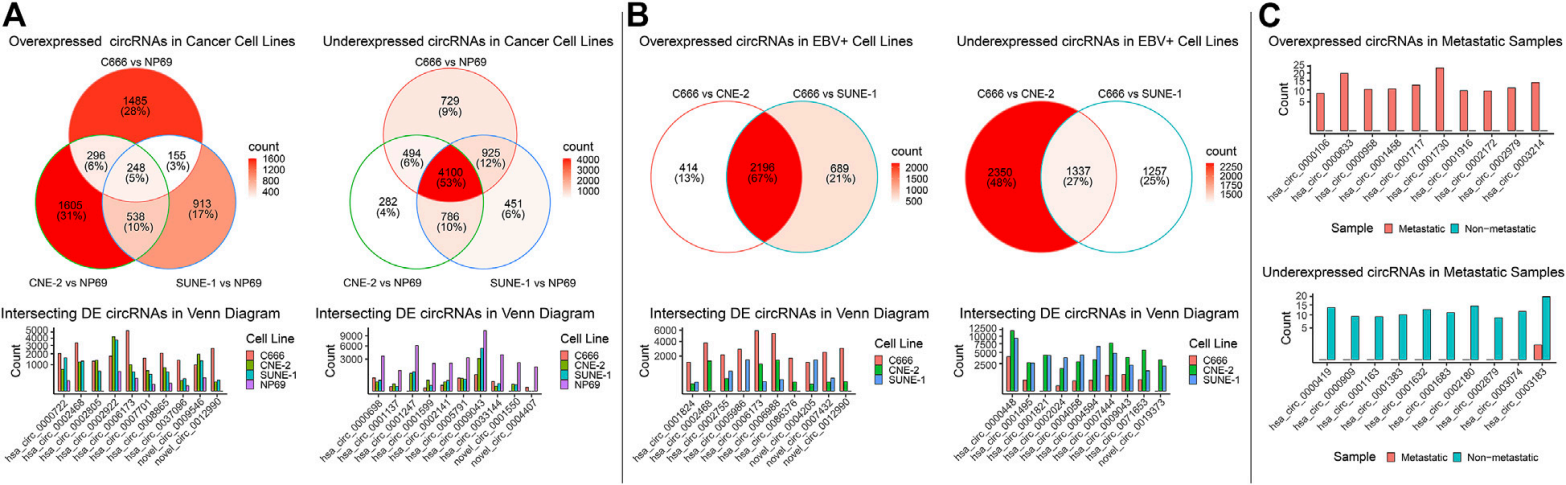
Overview of circRNA landscape across comparisons. The differential expression (DE) results for circRNAs across the three groups of comparison were integrated into one figure. **(A, C)** The expression of top intersecting DE-circRNA in each comparison was plotted. Functional enrichment was not performed as there were no commonly recognized tools for circRNA enrichment.

DE analysis on mRNA, miRNA, and circRNA was performed across four cell lines, falling under three groups of comparison: cancer (C666, CNE-2, SUNE-1) vs. non-cancer (NP69); EBV+ (C666) vs. EBV- (CNE-2, SUNE-1); and metastatic vs. non-metastatic using patient samples. In the cancer vs. non-cancer and EBV+ vs EBV- DE comparisons, where more than one set of DE analysis was performed, we focused on the intersecting differentially expressed mRNAs, miRNAs, circRNAs (DE-mRNAs, DE-miRNAs, DE-circRNAs). We plotted the top 10 overexpressed and under-expressed DE-RNA by log2 fold change. The summary of the DE analysis results across cell lines and samples for mRNA, miRNA, and circRNA was shown respectively in Figure 2; Figure 3; Figure 4. The entire DE analysis results can be found in Supplementary Table S1; Supplementary Table S2; Supplementary Table S3 which contains all DE analysis results of cancer vs. non-cancer cell lines, EBV+ vs. EBV- cell lines, metastatic vs. non-metastatic patient samples, respectively.

We then performed functional enrichment analysis on the intersecting set of DE-mRNA across each of the three groups of analysis (cancer, EBV, metastatic). We searched the GO Biological Pathways (BP), Molecular Functions (MF), and Cellular Component (CC) database using an over-representation test in the clusterProfiler R package (Wu et al., 2021). We highlighted functionally relevant and statistically significant pathways (Figures 2D–F). Similarly, we performed functional enrichment analysis on the intersecting set of DE-miRNA under each category of comparison. We used the miEAA website (Kern et al., 2020), searching across the GO, KEGG, and miRWalk (Sticht et al., 2018) databases. We highlighted functionally relevant and statistically significant pathways in Figures 3D–F. We then performed miRNA target gene enrichment using the miRTarBase (Huang et al., 2020) database, under the miEAA. The target enrichment results can be found in Supplementary Table S4.

### Enrichment of miRNA Target Genes and circRNA Source Genes

We performed target gene enrichment on the set of DE-miRNAs under each group of DE comparison. We utilized the miEAA (Kern et al., 2020) to perform Over-Representation Analysis (ORA), separately for the overexpressed and under-expressed DE-miRNAs to capture the directionality of mRNA regulation. Supplementary Figure S1 highlighted interesting functional enrichment results of DE-miRNA, and the full enrichment result can be found in Supplementary Table S4. In theory, the target genes for the set of overexpressed DE-miRNA would be down-regulated, and vice versa. Studies have shown that circRNAs regulate the expression of its source gene, such as circSEP3 and circSMARCA5 (Conn et al., 2017; Xu et al., 2020). To evaluate circRNA’s role as source gene regulators in NPC, we performed ORA using the KEGG database on the source genes of DE-circRNAs under each DE group (Supplementary Table S5).

### Target Prediction and Network Construction

**FIGURE 5:**
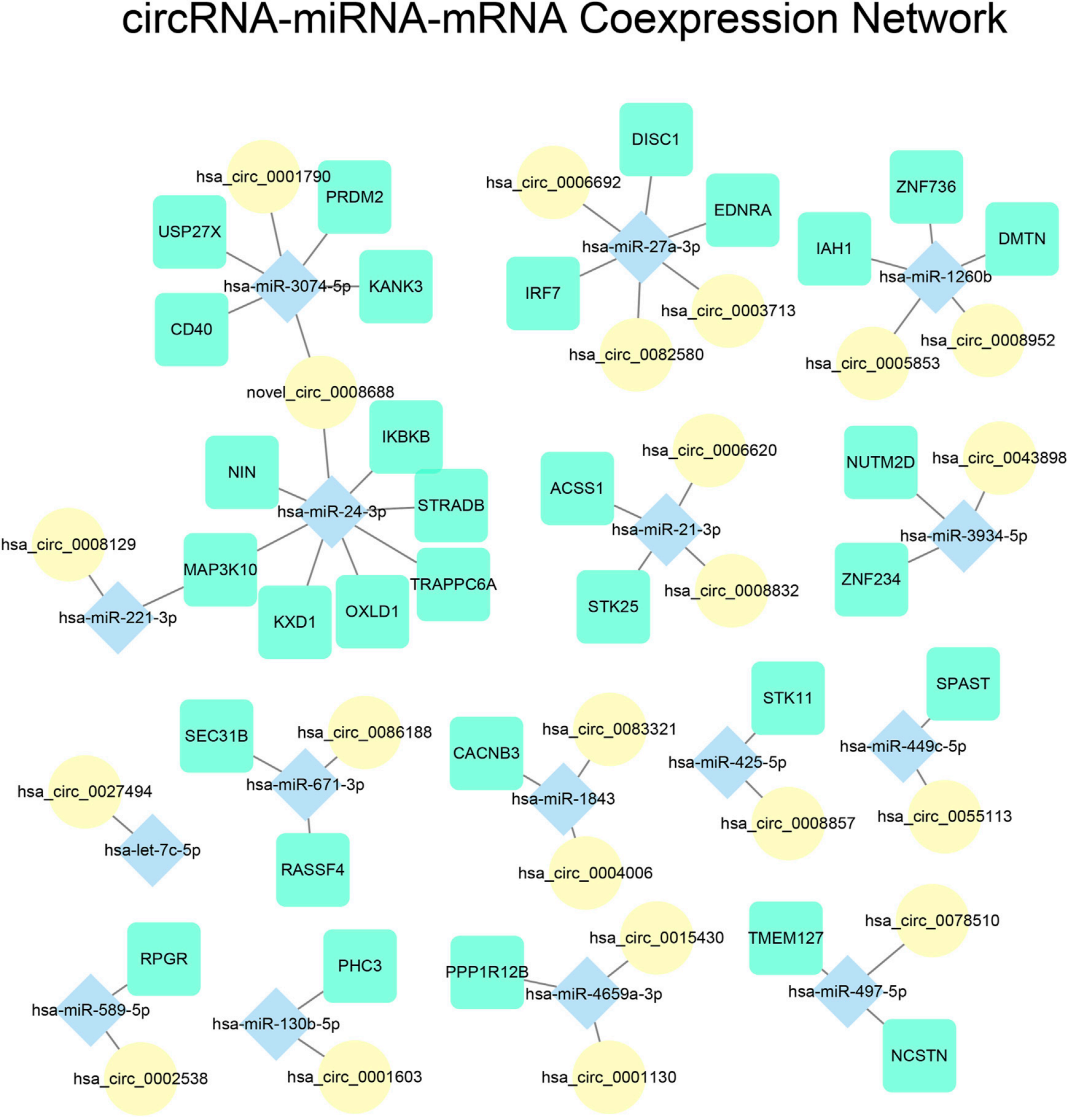
Visualization of circRNA-miRNA-mRNA network. Edges represent RNA pairs that have Pearson correlation coefficient < −0.85 and verified target interaction calculated by miRanda. mRNAs, miRNAs, and circRNAs were illustrated using different shapes and colors.

We constructed a circRNA-miRNA-mRNA co-expression network to investigate the role of ceRNA regulation in NPC. The Pearson correlation between all possible circRNA-miRNA and miRNA-mRNA pairs were calculated, and pairs with a correlation coefficient < −0.85 were considered to be significant. In efforts to reduce false positives, we used miRanda and its default parameters to determine whether the circRNA-miRNA and miRNA-mRNA pairs were valid targets, and removed pairs that were not deemed target pairs (Supplementary Table S6). Lastly, we filtered for miRNAs that were paired to at least one mRNA and circRNA to isolate complete ceRNA axes. The interaction network analysis yielded 428 miRNA-mRNA pairs, and 131 miRNA-circRNA pairs. We then extracted a subset of the network that contained only DE-miRNAs and their associated miRNAs and circRNAs, and visualized it using Cytoscape (Figure 5).

### Differentially Expressed circRNA-miRNA-mRNA Axes

The previous network analysis allowed us to gain an overview of the ceRNA landscape in NPC. However, not all elements in the network are implicated in NPC, therefore we leveraged DE analysis results to reveal the functionally relevant axes. From the previously constructed co-expression network, we extracted axes that contain at least one DE-circRNA and one DE-miRNA, and with opposite directions of differential expression between circRNA-miRNA and mRNA-miRNA pairs. In this process, we implicitly removed circRNAs that did not function as miRNA sponges, but instead regulate gene expression through other plausible routes such as binding RNA polymerases (Zhang et al., 2013), inducing methylation (Chen N. et al., 2018), or alternative splicing (Conn et al., 2017). These circRNAs are false positives, and will not show opposite differential expression to its predicted target miRNAs. In efforts to reduce false negatives, we included high scoring (TargetScore≥97) miRNA-mRNA pairs from the miRDB database (Chen and Wang, 2020). The result across three groups of DE comparisons: cancer (C666, CNE-2, SUNE-1) vs. non-cancer (NP69), EBV+ (C666) vs. EBV- (CNE-2, SUNE-1), and metastatic vs. non-metastatic patient samples, are shown in Table 1; Table 2; Table 3, respectively.

**TABLE 1:** DE circRNA-miRNA-mRNA axis in cancer vs non-cancer comparison.

Axis	RNA type	RNA ID	C666 vs NP69 Log2	SUNE-1 vs NP69 Log2	CNE-2 vs NP69 Log2
1	circRNA	hsa_circ_0002538	+1.55	+1.25	Not significant
	miRNA	hsa-miR-589-5p	−3.02	−1.10	−1.45
	mRNA	RPGR	+2.65	+1.48	+1.44
2	miRNA	hsa-miR-27b-3p	−2.87	−1.27	−1.35
	mRNA	TAB3	+1.23	+1.53	+1.25
3	miRNA	hsa-miR-20a-5p	+2.35	+2.03	+2.62
	mRNA	SAR1B	−2.12	−1.68	−1.66
	mRNA	BRMS1L	−1.13	−2.10	−1.61
4	miRNA	hsa-miR-493-5p	−5.60	−4.61	−5.76
	mRNA	ZIC2	+7.55	+7.80	+6.70
	mRNA	MBNL2	+1.91	+1.16	+0.90

**TABLE 2:** Differentially expressed circRNA-miRNA-mRNA axes in EBV+ vs EBV- cell lines.

Axis	RNA	RNA ID	C666 vs CNE-2 Log2FC	C666 vs SUNE-1 Log2FC
1	circRNA	hsa_circ_0027494	+9.23	+9.37
	miRNA	hsa-let-7c-5p	−2.60	−2.63
2	circRNA	hsa_circ_0005853	+10.27	+10.41
	circRNA	hsa_circ_0008952	+7.57	+1.69
	miRNA	hsa-miR-1260b	−4.06	−3.23
	mRNA	ZNF268	+3.06	+3.79
	mRNA	ZNF302	+10.07	+10.01
3	circRNA	hsa_circ_0008129	+8.89	+9.03
	miRNA	hsa-miR-221-3p	−3.86	−3.90
	mRNA	CDKN1B	+1.76	+1.17
	mRNA	RIMS3	+5.63	+3.57
4	circRNA	novel-circ-0008688	+3.21	+4.28
	circRNA	hsa_circ_0001790	+8.16	+8.29
	miRNA	hsa-miR-24-3p	−2.75	−2.60
	miRNA	hsa-miR-3074-5p	−2.76	−2.60
5	circRNA	hsa_circ_0055113	−9.22	−8.71
	miRNA	hsa-miR-449c-5p	+6.42	+7.28
6	circRNA	hsa_circ_0078510	−7.79	−7.60
	miRNA	hsa-miR-497-5p	+5.66	+5.11

**TABLE 3:** Differentially expressed miRNAs in metastatic vs non-metastatic patient samples.

RNA type	RNA ID	Log2FC	Adjusted *p* value
miRNA	hsa-miR-130b-5p	+2.03	0.00605
miRNA	hsa-miR-449c-5p	−6.93	1.09E-19
miRNA	hsa-miR-589-5p	+2.45	0.00204
miRNA	hsa-miR-671-3p	+1.82	0.0348

In Table 1, the differentially expressed circRNA-miRNA-mRNA axes in cancerous (C666, SUNE-1, CNE-2) vs. non-cancer (NP69) cell lines were listed. They must show significant differential expression between cancer and noncancerous cell lines (|log2 fold|≥ 1.0 and adjusted *p* value ≦ 0.05) and the miRNA must show negative correlation (correlation coefficient < −0.85) with predicted target by miRanda or miRDB. Adjusted *p* values and insignificant DE-mRNAs associations were omitted for visual clarity. The hsa_circ_103,862 is verified to bind miR-493-5p (Wang et al., 2020).

Similarly, in Table 2, the differentially expressed circRNA-miRNA -mRNA axis in EBV+ (C666) vs EBV- (CNE-2, SUNE-1) cell lines were listed. A positive value indicated higher expression in the C666 cell line and vice versa. They must show significant differential expression between EBV+ and EBV- cell lines (|log2 fold|≥ 1.0 and adjusted *p* value ≦ 0.05) and the miRNA must show negative correlation (correlation coefficient < −0.85) with predicted target by miRanda or miRDB. Insignificant DE-mRNAs associations and adjusted *p* values were omitted for visual clarity. CDKN1B, Ensemble ID ENSG00000111276, is verified to be a target of miR-221-3p *via* luciferase reporter assay (Fornari et al., 2008; Yin et al., 2019).

In Table 3, differentially expressed miRNAs in two metastatic vs. two non-metastatic patient samples were shown. A positive value indicated higher expression in the metastatic group and vice versa. Only miRNAs showing significant differential expression (|log2 fold| ≥ 1.0 and adjusted *p* value ≦ 0.05) were included.

From the list of circRNA-miRNA-mRNA axes, we identified the hsa_circ_0008129/miR-221-3p/CDKN1B axis, which was significantly differentially expressed in EBV+ (C666) vs. EBV- (CNE-2, SUNE-1) cell lines. We also highlighted the ceRNA axis behind *ZNF302, ZNF268, TAB3*, *SAR1B*, *BRMS1L, ZIC2*, and *MBNL2*.

### Differential Expression Analysis in Public Data

**FIGURE 6:**
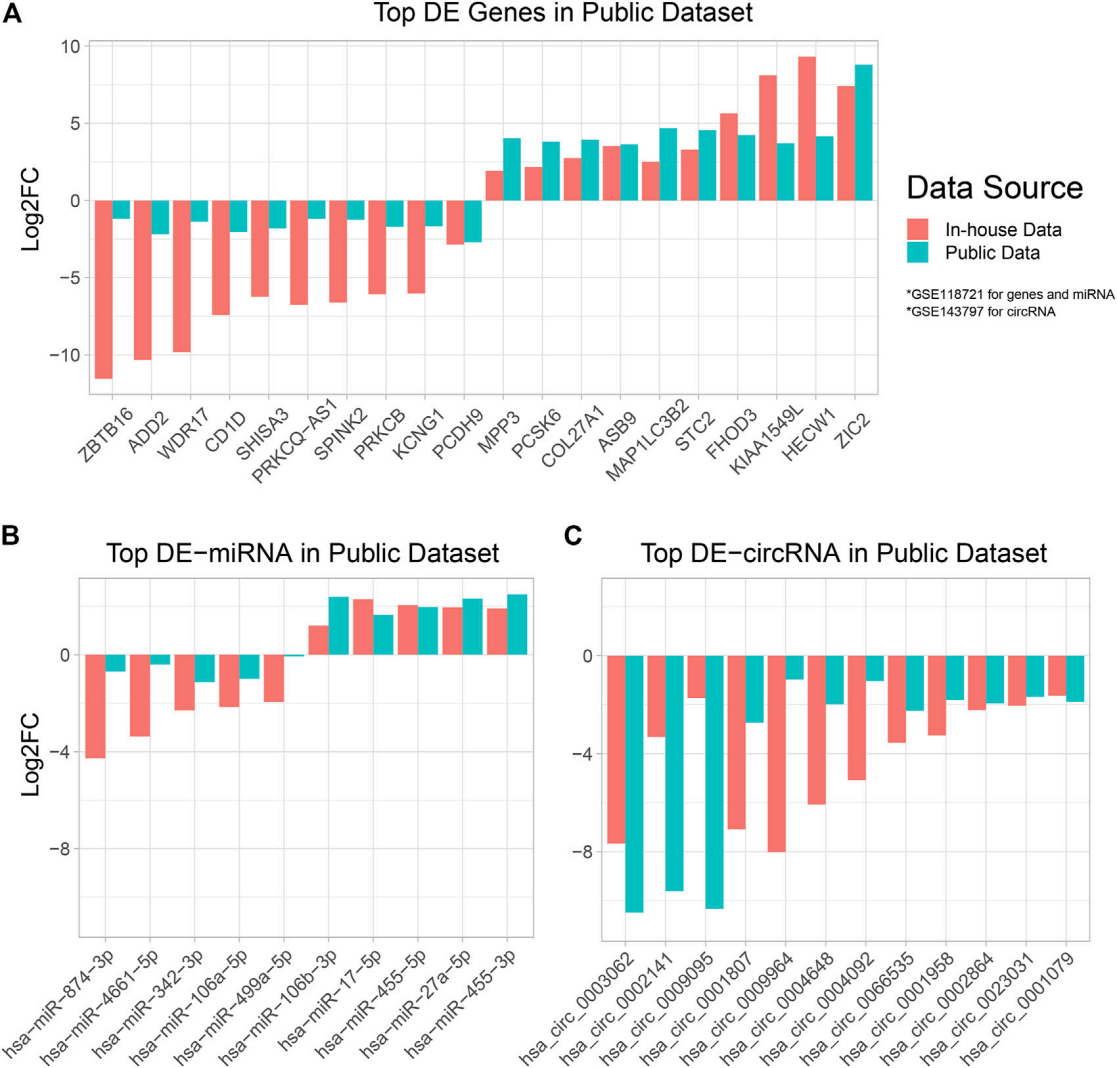
Bar plot of DE-RNAs in cross-referenced public datasets. The differentially expressed mRNA, miRNA, and circRNAs across two public datasets GSE118721 (Lin et al., 2018) and GSE143797 (Yang et al., 2020) were established and plotted alongside in-house data. A positive Log2FC denotes overexpression in tumor tissue, and a negative Log2FC denotes under-expression in tumor tissue. **(A,B)** DE performed using data from this study and GSE118721 (Lin et al., 2018). **(C)** DE performed using data from this study and GSE143797 (Yang et al., 2020).

To verify our computational findings and highlight promising DE-RNAs, we utilized two additional public datasets: GSE143797 for circRNA expression in NPC and normal tissue (Yang et al., 2020), GSE118721 for miRNA and mRNA expression in NPC and normal tissue (Lin et al., 2018). We performed a Two-way Student T test to test for differentially expressed RNAs in tumor verses normal tissue, and reported genes with adjusted *p* value ≦ 0.05 and whose direction of differentiation aligns with our data (Supplementary Table S7). We plotted the most highly differentially expressed mRNA, miRNA, and circRNA, and their respective fold changes in data from this study and prior ones (Figure 6).

## Discussion

The role of ceRNAs in inducing the epigenetics changes necessary for the development of tumors is a topic of great research interest. The ceRNA network method is an efficient method to capture the complexity of interactions between a diverse pool of ceRNAs, and different studies have demonstrated its effectiveness in discovering important epigenetic changes in cancer. Specifically, this method allows the emerging role of circRNAs as both miRNA sponges and direct transcription regulators to be integrated into the omics landscape of cancer.

In this study, we profiled the expression of mRNAs, miRNAs, and circRNAs in NPC cell lines and non-cancerous human immortalized nasopharyngeal epithelial cell line. We utilized the differential expression of mRNAs, miRNAs, circRNAs, and the construction of a co-expression network to identify differentially expressed circRNA-miRNA-mRNA axes in NPC. Many of the axes we found contain well-established oncogenic miRNAs, and potentially point to post-transcriptional regulatory events in NPC. We found convincing evidence for the existence of a *hsa_circ_0002538/miR-589-5p/RPGR* axis dysregulated in NPC, and a *hsa_circ_0008129/miR-221-3p/CDKN1B* axis, which is differentially regulated in EBV+ vs. EBV- cell lines. Again, the final collection of axes underwent a stringent set of filters, namely, the RNA components have to: negatively co-expressed with correlation coefficient < −0.85, exhibit significant DE in its respective group, and predicted to interact by the miRanda software or miRDB database. It is nevertheless important to keep in mind that cell culture often drift from its ancestral expression profiles, therefore our findings warrant further validation using ideally fresh tissue samples.

### Cancer vs. Noncancer Cell Lines: Differentially Expressed circRNA -miRNA-mRNA Axes

We have found evidence in Axis 1 (Table 1) for a potential hsa_circ_0002538↑miR-589-5p↓RPGR↑ axis. miR-589-5p is a well-established cancer-associated miRNA, found to inhibit *MAP3K8* in hepatocellular carcinoma (Zhang et al., 2016), regulate tumor growth in HCC by targeting *MIG-6* (Xu et al., 2018), and act as tumor suppressor in prostate cancer (Ji et al., 2019). Alongside the under-expressed miR-589-5p, we observe an overexpression of hsa_circ_0002538, predicted to bind to miR-589-5p. We postulate that dysregulation of hsa_circ_0002538 is the source of downstream abnormalities in expression. *RPGR* encodes the retinitis pigmentosa GTPase regulator, whose function in NPC and cancer is unknown. Given that other various GTPases are well known in their regulatory roles in cancer, it is plausible that RPGR is associated with NPC progression (Fernández-Medarde and Santos, 2011; Prieto-Dominguez et al., 2019; Boudhraa et al., 2020; Clayton and Ridley, 2020). It is also possible that *RPGR* is simply a passenger event in the axis.

Furthermore, we identified three miRNA-mRNA axes, for which no DE-circRNA component was found in our analysis. In Axis 2 (Table 1 **)**, miR-27b-3p↓TAB3↑, over-expression of TAB3 has been found to promote tumor progression in NSCLC (Chen et al., 2016), colorectal cancer (Luo et al., 2017), and triple-negative breast cancer (Tao et al., 2016). Under-expression of miR-27b-3p has been shown to induce drug resistance in breast cancer (Chen D. et al., 2018). In Axis 3 (Table 1 **)**, miR-20a-5p↑ SAR1B↓ BRMS1L↓, reduced BRMS1L in breast cancer tissues was shown to be associated with metastasis and poor patient survival (Gong et al., 2014). Specifically, Gong et. Al. found that BRMS1L inhibited epithelial-mesenchymal transition, and thus inhibiting breast cancer metastasis. In Axis 4 (Table 1 **)**, miR-493-5p↓ ZIC2↑ MBNL2↑, MBNL2 is abnormally expressed in lung and breast cancer (Zhang J. et al., 2019), as well as hepatocellular carcinoma (Lee et al., 2016). Interestingly, despite overexpressed in these cancer types, both studies showed that MBNL2 suppresses tumor progression. ZIC2 was shown to be highly overexpressed in NPC both in the data used in this study, as well as in a study by Lv et al. (Lv et al., 2021) and Lin et al. (Lin et al., 2018).

### EBV+ vs. EBV- Cell Lines: Differentially Expressed circRNA-miRNA -mRNA Axes

In comparison to few complete axes in the cancer vs non-cancer comparison, the EBV+ (C666) vs. EBV- (CNE-2, SUNE-1) comparisons revealed several axes of circRNA-miRNA exhibiting differential expression. Surveying through the DE results of target miRNA-circRNA pairs, we observed that the directions of differential expression are almost uniformly opposite, which confirms the theory that circRNAs serve as miRNA sponges.

In Axis 3 (Table 2 **)** we observed hsa_circ_0008129↑miR-221-3p↓*CDKN1B*↑*RIMS3*↑ in EBV+ (C666) cell line. Prior studies confirmed that miR-221-3p targets the cell cycle regulator *CDKN1B*, also known as p27, through luciferase reporter assay (Fornari et al., 2008; Yin et al., 2019). *CDKN1B* encodes the cyclin dependent kinase inhibitor 1B protein, belonging to the Cip/Kip protein family. CDKN1B functions as a cell cycle check point at G_1_-S by repressing cyclin-dependent kinases that are necessary for progression from G_1_ to S phase (Sherr and Roberts, 1999). Overall, *CDKN1B* is mainly dysregulated at the post-transcriptional level in human cancer, which supports our finding of a significantly differentially expressed ceRNA axis. Further, *CDKN1B* is described as haplo-insufficient tumor suppressor gene, where animals lacking one copy of *CDKN1B* already displayed tumor-prone phenotypes (Fero et al., 1998). RIMS3 is not reported to be associated with cancer to the best of our knowledge, and was likely a passenger event.

The role of the miRNA component of this axis, miR-221-3p, has been studied extensively across cancer types. A study by Wang et al. showed that miR-221-3p serve as potential prognostic predictors for hepatocellular carcinoma (Wang et al., 2019). Other studies have shown miR-221-3p to be involved in drug resistance in glioma cells (Milani et al., 2019) and breast cancer (Chen et al., 2020) by increasing antiapoptotic abilities. There have been no studies done on hsa_circ_0008129 to the best of our knowledge. Our results show evidence for a *hsa_circ_0008129/miR-221-3p/CDKN1B* axis, which potentially plays a role in explaining the pathological differences between EBV+ and EBV- NPC.

In Axis 2 (Table 2 **)**, we observed an overexpression of miR-1260b in all EBV- cell lines. MiR-1260b is a well-studied oncogenic miRNA, and has been found to regulate proliferation in non-small cell lung carcinoma (NSCLC) (Xu et al., 2015; Xia et al., 2019) and prostate cancer (Hirata et al., 2014). *ZNF268* is predicted to be a high affinity target of miR-1260b, with a target score of 100 (the maximum of target score) in the miRDB database (Chen and Wang, 2020), and is found to be overexpressed in cervical cancer (Wang et al., 2012) and ovarian carcinomas (Hu et al., 2013). Specifically, Wang et al. found that knockdown of *ZNF268* in cervical cancer cells caused cell cycle arrest at the G0/G1 phase (Wang et al., 2012). Similarly, *ZNF302* is predicted to be a miR-1260b target gene (score = 98), and high expression of *ZNF302* is associated with poor survival in Endometrial Carcinoma in TCGA-UCEC (Supplementary Figure S2). We found both *ZNF268* and *ZNF302* to be overexpressed in EBV + cell line C666, which may explain why there is higher malignancy in the EBV + subtype of NPC. Alongside miR-1260b and zinc finger proteins, we observed up-regulation of hsa_circ_005853 and hsa_circ_0008952 in the EBV+ subtype of NPC., which were predicted to bind miR-1260b. These circRNAs have not been studied to the best of our knowledge. The above results suggest that the Axis 2 (Table 2) plays a key role in the progression of NPC and warrants further studies.

In Axis 1, 4, 5, 6 (Table 2 **)**, we observed abnormal expression of various circRNA-miRNA pairs, some of which were previously found to be associated with cancer. In Axis 1 (Table 2 **)**, has_circ_0027494↑ let-7c-5p↓, let-7c-5p is found to be a tumor suppressor across many cancer types, including breast cancer (Fu et al., 2017), mucosal melanoma (Tang et al., 2019), acute erythroleukemia (Mortazavi and Sharifi, 2018), among others (Wagner et al., 2014; Liu et al., 2018; Chirshev et al., 2019). We postulate that hsa_circ_0027494 has inhibitory effects on let-7c-5p (Panda, 2018; Zhang P.-F. et al., 2019; Zhang X. et al., 2019), leading to downstream overexpression of let-7c-5p targeted genes, which could explain tumorigenic activities that stands differently in EBV + vs EBV- NPC. Similarly, for Axis 4, 5, 6 (Table 2 **)**, it is plausible that they regulate certain target genes that underlies traits of EBV+ NPC.

In sum, our method combining DE analysis and co-expression network analysis yielded 6 circRNA-miRNA pairs that show significant DE and co-expression. Out of these results, we were able to establish the *hsa_circ_0008129*↑ *miR-221-3p*↓ *CDKN1B*↑ axis, where overexpression of hsa_circ_0008129 led to the sponging of miR-221-3p and upregulation of *CDKN1B*.

### Metastatic vs. Non-metastatic Patient Samples: Differentially Expressed mRNAs

No significant mRNA or circRNA differential expression was detected in the differential expression analysis of metastatic vs. non-metastatic samples. However, we did find 4 miRNAs that were differentially expressed in the metastatic samples than the non-metastatic samples (Table 3).

miR-130b-5p was overexpressed in the metastatic samples. This miRNA is likely not involved in pan-cancer metastatic mechanisms, as different cancer types exhibit different expression patterns with contradicting effects. Namely, miR-130b-5p has been shown to promote proliferation and migration in gastric cancer *via* targeting *RASAL1* (Chen H. et al., 2018), as well as in osteosarcoma *via* binding to *TIMP2* (Cheng et al., 2019). On the other hand, miR-130b-5p exhibits anti-tumor effects in pancreatic ductal adenocarcinoma (Fukuhisa et al., 2019) and prostate cancer (Chen et al., 2015). Little literature exists on the functions of miR-449c-5p (Table 3), yet given its significant downregulation in the metastatic group (Log2 = -6.9275, *p* = 1.0903E-19), we postulated that miR-449c-5p could target a metastasis-associated mRNA.

## Materials and Methods

### Data Source

We used four cell lines, including three nasopharyngeal carcinoma (NPC) cell line: SUNE-1, C666 (EBV+), CNE-2, and one non-cancerous human immortalized nasopharyngeal epithelial cell line NP69. Furthermore, we collected samples from four patients diagnosed with NPC in Fujian Cancer Hospital. Two of the four NPC patients were grouped into the metastatic group, and the remaining two patients were grouped into the non-metastatic group.

### Statistics

All adjusted *p* values were reported at false discovery rate of 0.05 unless otherwise specified. All measures of correlation referred to Pearson correlation unless otherwise specified.

### Sequencing and Preprocessing

We used Hisat2 to align the RNA reads to the reference genome (Kim et al., 2019). We used StringTie (https://github.com/gpertea/stringtie) (Pertea et al., 2015) to perform RNA transcript assembly. After RNA transcript assembly, we used cuffmerge in the Cufflinks software (Trapnell et al., 2010) to filter the transcript, namely by removing transcripts smaller than 200 nt, or have less than 2 exons, or transcripts with unidentified directions. Then we referenced the remaining transcript assembly using cuffcompare to select for known circRNA, mRNA, and miRNAs. We used StringTie to profile the expression of different RNAs given the filtered transcript data. Information related to QC and mapping can be found in Supplementary Table S8.

### Identification of circRNAs

We used two software to identify the circRNAs within the transcript assembly: find_circ (Memczak et al., 2013) and CIRI2 (Gao et al., 2015; Gao et al., 2017). Each software produced a set of identified circRNAs, and we took the intersecting set between both to minimize false positive rates. We profiled the expression of circRNAs using CIRI2. We obtained the expression of 15,036 circRNAs, out of which 7,029 were novel circRNAs found in this study. The complete list of all circRNAs identified in this study can be found in Supplementary Table S9.

### Differential Expression Analysis

We performed DE analysis for miRNA, circRNA, and mRNA separately among different pairs of cell lines using DESeq2 (Love et al., 2014). Namely, we performed DE analysis on each category of comparison: cancer (C666, CNE-2, SUNE-1) vs. non-cancer (NP69); EBV+ (C666) vs. EBV- (CNE-2, SUNE-1); and an additional set of DE analysis using two metastatic vs. two non-metastatic patient samples. The threshold for qualifying as differential expression was set to be edgeR adjusted *p* value ≦ 0.05 and |log2FoldChange| ≥ 1.0.

### Network Construction

To investigate the role of ceRNA regulation in NPC, we constructed a co-expression network representing the interactions between miRNA-circRNA pairs and miRNA-mRNA pairs. We first calculated Pearson correlation between all possible miRNA-circRNA and miRNA-mRNA pairs, and keep only pairs with correlation coefficient < −0.85. The specific cutoff was chosen to capture the biological mechanism where circRNA inhibits miRNA and miRNA inhibits mRNA. Further, we used the miRanda software (Betel et al., 2010) to predict miRNA-circRNA and miRNA-mRNA target interactions, and we kept only pairs that were calculated to be valid targets by the default parameters of miRanda. Due to the limited number of cell lines for co-expression calculation, and potential flaws in the miRanda software, we further curate the list of miRNA-mRNA pairs by adding those with target score≥97 in the miRDB database (Chen and Wang, 2020). The target score are assigned by the target prediction algorithm in miRDB and the higher the target score is, the more confidence the prediction has (Chen and Wang, 2020). It ranged from 50 to 100. The target scores of miRNA-mRNA pairs in Table 1; Table 2 were given in Supplementary Table S10.

### Identifying Significant circRNA-miRNA-mRNA Axes

In order to identify functionally relevant axes of regulation between circRNA-miRNA-mRNA, we applied additional filters specifying negative correlations. Namely, we selected circRNA-miRNA-mRNA axes in the network satisfying: 1) contained at least 2 differentially expressed components; 2) circRNA-miRNA and miRNA-mRNA pairs within the axis exhibited opposite differential expression. The motivation behind such criteria was that given a functionally relevant gene with abnormal expression, the upstream circRNAs and miRNAs, if indeed responsible for the abnormal expression, should have opposing directions of DE. Namely, miRNAs inhibit expression of target gene using Dicer and Drosha protein complexes, and circRNAs inhibit target miRNA through the miRNA sponge mechanism. Therefore, within a relevant axis, miRNAs should be negatively correlated to circRNAs and mRNAs.

### DE of Public Datasets

GSE143797 profiled the expression of four NPC tissue and matched healthy tissue, identifying 93 upregulated circRNAs and 77 downregulated circRNAs (Yang et al., 2020). GSE118721 profiled the mRNA and miRNA expression of six EBV-positive NPC biopsy specimens and normal nasopharyngeal samples (Lin et al., 2018). We performed a Two-way Student T test to identify differentially expressed coding and non-coding RNAs both datasets, and calculated Log2 Fold Changes.

### Kaplan-Meier Plots of Genes of Interest

The GEPIA portal (Tang et al., 2017) (gepia.cancer-pku.cn) was used to plot Kaplan-Meier curves for genes of interest. Appropriate TCGA datasets are used, and the cutoff for high and low expression is the top and bottom 25th percentile. Overall survival was plotted alongside Hazard Ratio and the 95% confidence interval.

## Conclusion

Our study compared the expression profile between NPC vs. non-cancerous human immortalized nasopharyngeal epithelial cell lines, EBV+ vs. EBV- NPC cell lines, and metastatic vs. non-metastatic patient samples. Specifically, we constructed a circRNA-miRNA-mRNA co-expression network, and performed differential expression analysis. This allowed us to filter for highly correlated non-coding RNA axis that show significant differential expression, suggesting that they are both biologically linked and significant in the genetic landscape of NPC. We found multiple circRNA-miRNA-mRNA axes relevant in both NPC and more specifically in EBV+ NPC. We highlighted the regulatory ceRNA axes behind key differentially expressed genes including *CDKN1B, ZNF302, ZNF268, TAB3*, *SAR1B*, *BRMS1L, ZIC2*, and *MBNL2*. We believe our in-silico findings illustrated the regulatory role that ceRNAs play in NPC, and these results should be further studied using experimental techniques.

## Data Availability

The datasets presented in this study can be found in online repositories. The names of the repository/repositories and accession number(s) can be found in the article/Supplementary Material.
