# Supplementary material for: Competitive Endogenous RNA Landscape in Epstein-Barr Virus Associated Nasopharyngeal Carcinoma
Source: Front Cell Dev Biol. 2021 Nov 4;9:782473. doi: 10.3389/fcell.2021.782473 (PMC8600047; doi:10.3389/fcell.2021.782473)

Target Enrichment of DE-miRNAs

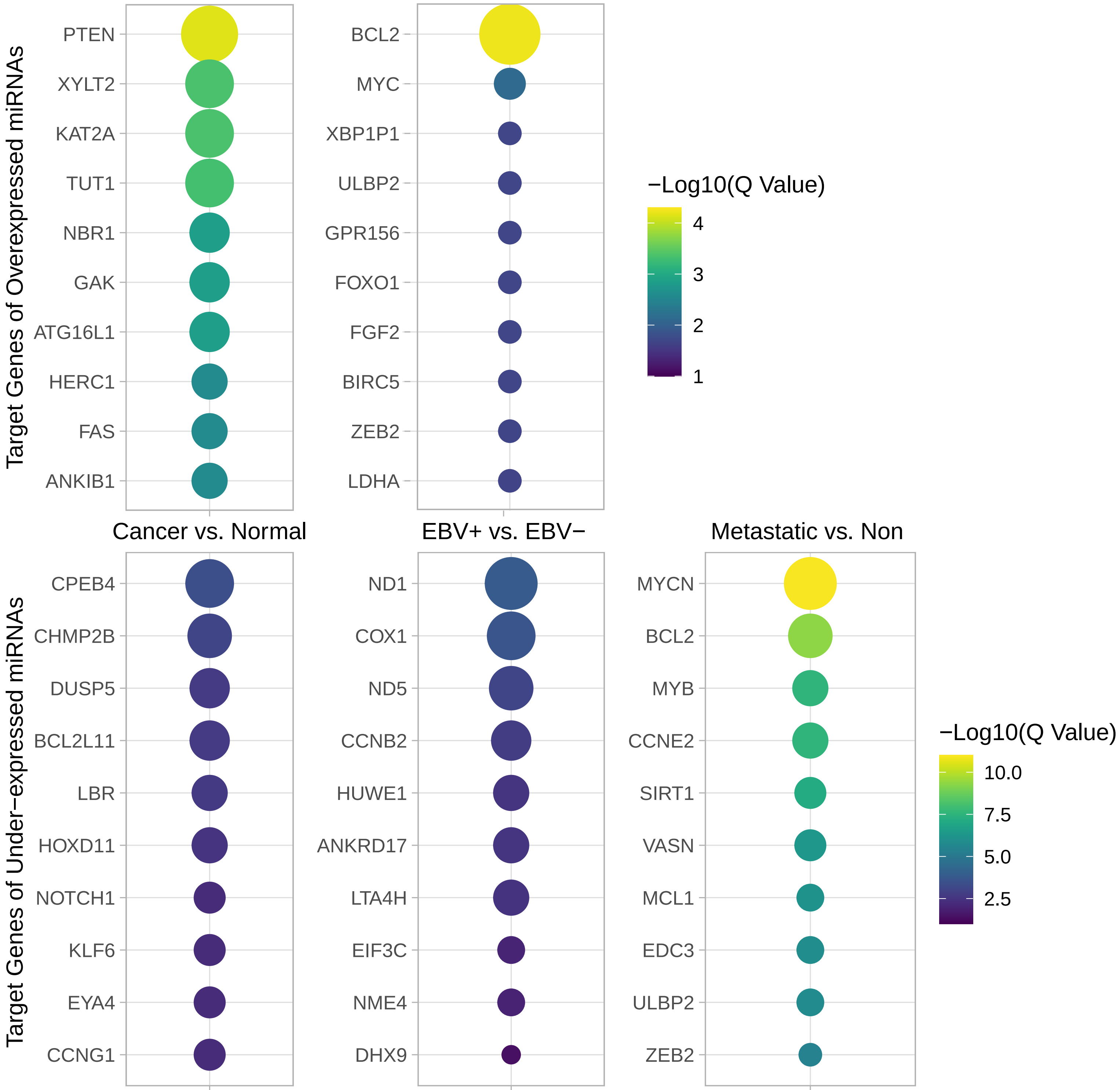

Supplement: Supplementary file 6 [file DataSheet1.PDF]
